# Increased NK cell immunity in a transgenic mouse model of NKp46 overexpression

**DOI:** 10.1038/s41598-017-12998-w

**Published:** 2017-10-12

**Authors:** Ariella Glasner, Batya Isaacson, Sergey Viukov, Tzahi Neuman, Nehemya Friedman, Michal Mandelboim, Veronika Sexl, Jacob H. Hanna, Ofer Mandelboim

**Affiliations:** 1 The Lautenberg Center for General and Tumor Immunology, Department of Immunology and Cancer Research, IMRIC, Faculty of Medicine, The Hebrew University Medical School, Jerusalem, Israel; 2 Department of Molecular Genetics, Weizmann Institute of Science, Rehovot, 7610001 Israel; 3 Department of Pathology, Hadassah Medical Organization, The Hebrew University Medical Center, Jerusalem, Israel; 4 Central Virology Laboratory, Ministry of Health, Chaim Sheba Medical Center, Tel-Hashomer, Ramat-Gan, Israel; 5 Department of Epidemiology and Preventive Medicine, School of Public Health, Sackler Faculty of Medicine, Tel-Aviv University, Tel-Aviv, Israel; 6 Institute of Pharmacology and Toxicology, University of Veterinary Medicine, Vienna, Austria

## Abstract

Natural Killer (NK) cells employ activating receptors like the Natural Cytotoxicity Receptors (NCRs: NKp30, NKp44 and NKp46), of which only NKp46 has a mouse orthologue (Ncr1), to eliminate abnormal cells. NKp46/Ncr1 is considered a selective marker for NK cells, although it is also found on a subset of ILCs, where it appears to be without function. The influenza virus hemagglutinin (HA) was the first ligand identified for Ncr1/NKp46 followed by other viral, bacterial and even fungal ligands. NKp46/Ncr1 also recognizes unknown self and tumor ligands. Here we describe the generation of a transgenic mouse where the Ncr1 gene is expressed in the Rosa locus, preceded by a floxed stop sequence allowing Ncr1/NKp46 expression in various tissues upon crossing with Cre transgenic mouse lines. Surprisingly, while several crossings were attempted, Ncr1 overexpression was successful only where cre recombinase expression was dependent on the Ncr1 promoter. Ncr1 overexpression in NK cells increased NK cell immunity in two hallmark Ncr1 related pathologies, influenza virus infection and B16 melanoma. These data suggest that increasing NK cell cytotoxicity by enforced NKp46/Ncr1 expression serves as a potential therapeutic opportunity for the treatment of various pathologies, and in immunotherapy.

## Introduction

NK cells are important first line of defense innate lymphocytes. They were shown to participate in many immunological and regulatory processes including viral^1–11^, bacterial and fungal infections^12–14^, cancer^8,9,15–20^, graft versus host disease^21^, autoimmunity^22–24^, allergy^25^ and pregnancy^26^. NK cells kill their targets when signals from activating and co-activating receptors overcome inhibitory signals^27^. Inhibitory NK receptors recognize MHC class I molecules^28^, but also non-MHC-I ligands such as CEACAM and PVR^29–33^. Among the activating receptors are NKG2D, and the NCRs: NKp30, NKp44 and NKp46. The ligands for these receptors are frequently induced by stress (mainly for NKG2D)^34–38^, or are pathogen-derived (mainly for the NCRs). Examples include the HA of influenza, and other viruses^10,39^, that are recognized by all three NCRs and the EPA 1,6 and 7 of *Candida glabrata* that are recognized by NKp46^14^. NK cells also recognize ligands expressed on the surface of normal (e.g. pancreatic or hepatic^22,23^) or malignant cells, whose identity is still unknown^8,17^.

NKp46 has a key role among NK cell activating receptors. It is expressed on all NK cells and is the only NCR present in mice (Ncr1). Using an Ncr1 knockout (KO) mouse (Ncr1^*gfp/gfp*8^), we and others have shown the involvement of Ncr1 in influenza virus and human metapneumovirus (hMPV) infections^6–9,18,40,41^. We also demonstrated the involvement of NKp46 in modulating cancer development and metastasis^8,16,17,20^, graft versus host disease^21^, type I and Type II diabetes^24,42^, liver fibrosis^22^, bacterial^12,13,43^ and candida glabrata infection^14^, underscoring the privileged and unique role of this receptor.

Several mice deficient for other NK activating receptors were generated, including NKG2D deficient mice^44,45^, and a mouse lacking both NKG2D and NKp46^46^. NK cells in these mice developed normally, with some exceptions where moderate alterations in NK cell receptor repertoire were observed^45^. To gain further insights on NKp46 activities we now generated an “Ncr1 gain of function” mouse (denoted Ncr1^cre^ Ncr1^OE^) that enables the overexpression of Ncr1 specifically in NK cells.

## Results

### Generation of a Rosa26^*Flox-Stop-Flox-IRES-Ncr1*^ transgenic mouse

**Figure 1:**
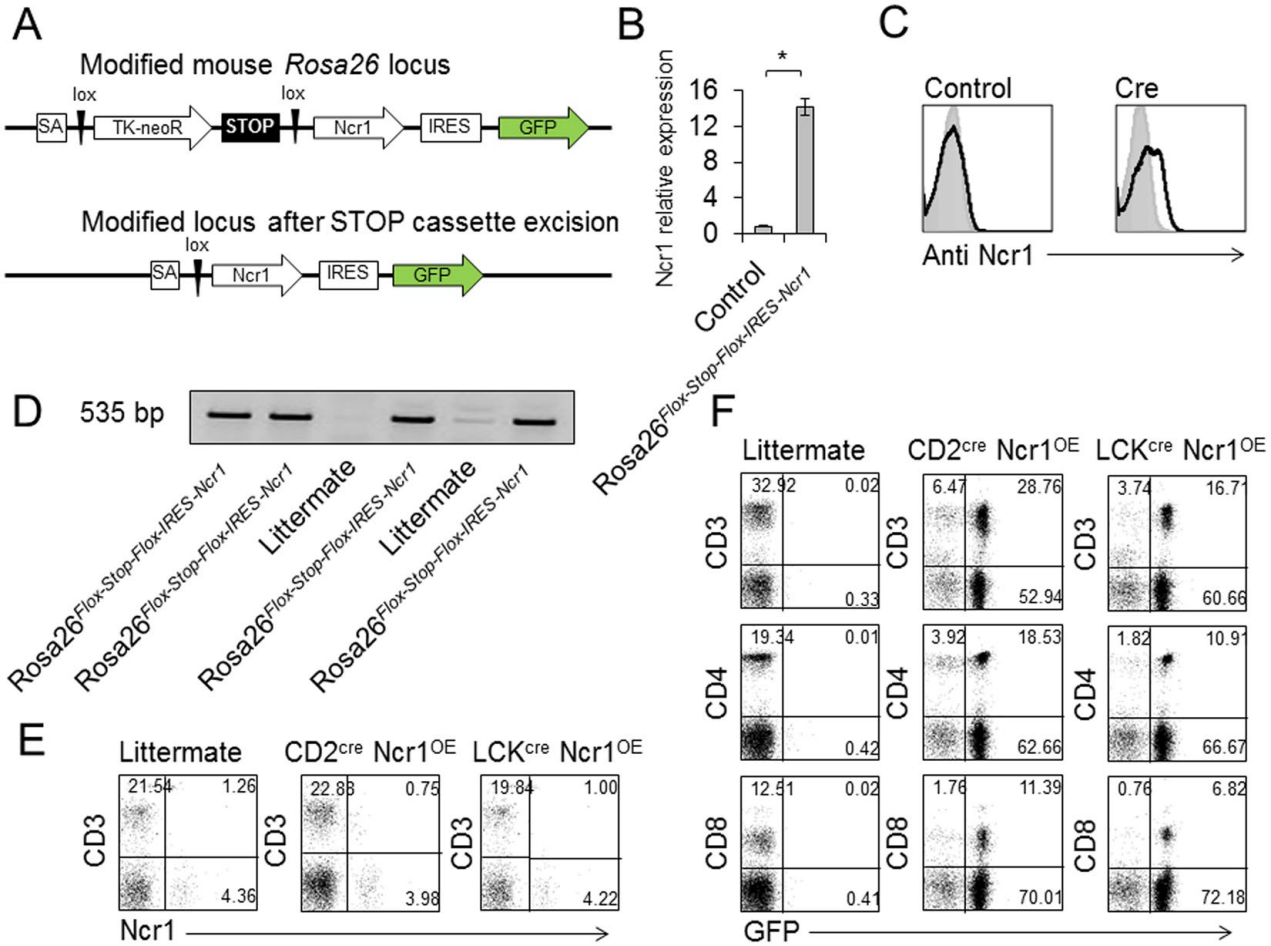
Generation of Rosa26^*Flox-Stop-Flox-IRES-Ncr1*^ mouse and crossing with CD2^cre^ and LCK^cre^ mice. (**A**) Schematic representation of the Rosa locus-targeting vector. (**B**) Clones of ES cells were tested for Ncr1 mRNA expression following cre recombinase treatment. Ncr1 relative expression, compared to control ES cells is presented. The experiment combines data from three independent experiments. Values are shown as mean ± SEM. *P < 0.05. (**C**) FACS staining of ES cells, untreated, or following cre treatment. Gray histograms represent background control. Black histograms represent specific staining. Each FACS plot is representative of at least three independent experiments. (**D**) Chimeric mice were crossed to WT C57BL/6 mice and the progeny were genotyped using specific primers. DNA samples run on gel are presented. Specific bands sized 535 bp signify the inserted Ncr1 gene. The figure represents the four founder breeders. Contrasts were adjusted for clarity. (**E,F**) Rosa26^*Flox-Stop-Flox-IRES-Ncr1*^ mice were bred with CD2^cre^ and LCK^cre^ mice. Offspring were denoted CD2^cre^ Ncr1^OE^ and LCK^cre^ Ncr1^OE^ respectively. (**E**) FACS plots depicting CD3 and Ncr1 expression in the transgenic mice, compared to a littermate. (**F**) FACS plots depicting CD3, CD4 and CD8, and GFP expression in the transgenic mice, compared to a littermate. The plots are representative of five independent stainings. Percentages are indicated.

To study the consequences of enforced expression of Ncr1 *in vivo* we generated a mouse denoted Rosa26^*Flox-Stop-Flox-IRES-Ncr1*^. This mouse enables the expression of Ncr1 in any tissue, following crossing with cre recombinase expressing mouse strains. To generate the Rosa26^*Flox-Stop-Flox-IRES-Ncr1*^ mouse, we used the STOP-eGFP-ROSA26TV vector. Figure 1A depicts the original construct. Upon crossing with a cre recombinase expressing mouse, the stop cassette is excised and Ncr1 is expressed (Fig. 1A). We transduced the targeting vector into V6.5 mouse embryonic stem (ES) cells, and confirmed the correct insertion of the construct by qRT PCR (Fig. 1B), and by FACS staining of the ES cells following cre treatment (Fig. 1C). Clones of the *Ncr1* positive ES cells were injected to blastocyst stage embryos, and implemented in foster mothers. The chimera offspring were bred with wild type (WT) C57BL/6 mice, and four progeny carrying Ncr1 were selected for further breeding. Figure 1D shows the genotyping of the four Ncr1 expressing founders.

### Ncr1 overexpression in the T cell lineage

Our initial aim was to express Ncr1 in T cells to generate enhanced killer cells possessing both adaptive and innate properties. To our surprise, when crossing the Rosa26^*Flox-Stop-Flox-IRES-Ncr1*^ mice with either CD2^cre^ (CD2^cre^ Ncr1^OE^) or LCK^cre^ (LCK^cre^ Ncr1^OE^) mice, Ncr1 was not expressed on the cell surface (Fig. 1E). While analyzing the CD2^cre^ Ncr1^OE^ and LCK^cre^ Ncr1^OE^ T cells, we detected normal percentages of CD3, CD4 and CD8 expressing populations in the transgenic mice (Fig. 1F). GFP expression (part of the construct, Fig. 1A) was evident in about three quarters of the T cells. A large population of Non-T cells was also found to express GFP (Fig. 1F), including B cells and other immune cells, however none of these cells expressed Ncr1.

### Characterization of the Ncr1^cre^ Ncr1^OE^ NK cells

**Figure 2:**
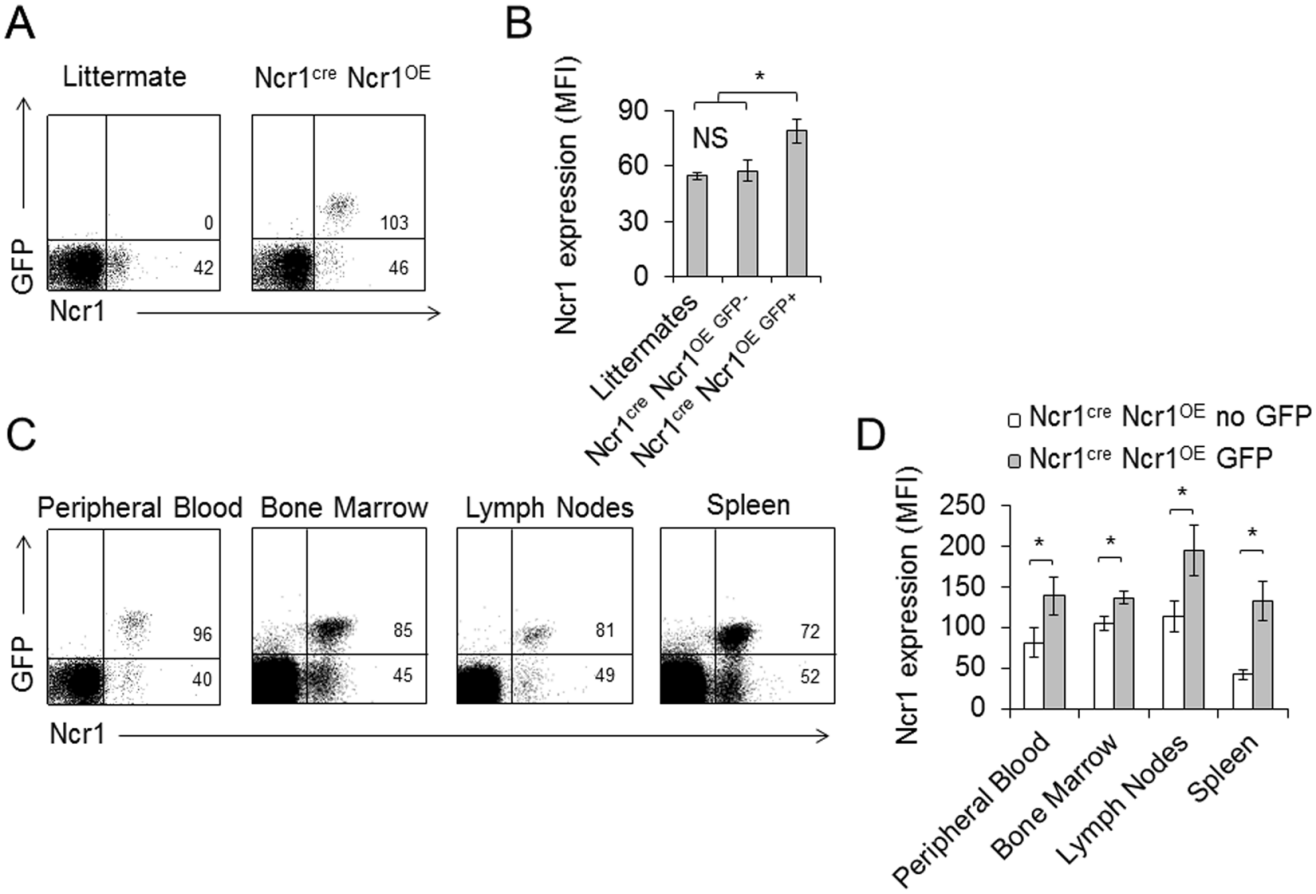
Ncr1 is overexpressed in Ncr1^cre^ Ncr1^OE^ mice NK cells. (**A,B**) Rosa26^*Flox-Stop-Flox-IRES-Ncr1*^ mice were bred with Ncr1^cre^ mice. Offspring were denoted Ncr1^cre^ Ncr1^OE^. (**A**) FACS plots depicting GFP and Ncr1. A TG and littermate control is shown. The plots are representative of at least five independent stainings. MFIs are indicated. (**B**) Quantification of the Ncr1 MFIs in the various indicated mice. Forty two littermates and 21 Ncr1^cre^ Ncr1^OE^ TGs were used. Values are shown as mean ± SEM. *P < 0.05. NS-Non-significant. (**C**) FACS plots depicting GFP and Ncr1. A TG and littermate control is shown. The plots are representative of five independent stainings. MFIs are indicated in the quadrants. (**D**) Quantification of the Ncr1 MFIs in the various compartments in each cell population. At least six littermates and six Ncr1^cre^ Ncr1^OE^ transgenes were used. Values are shown as mean ± SEM. *P < 0.05.

**Figure 3:**
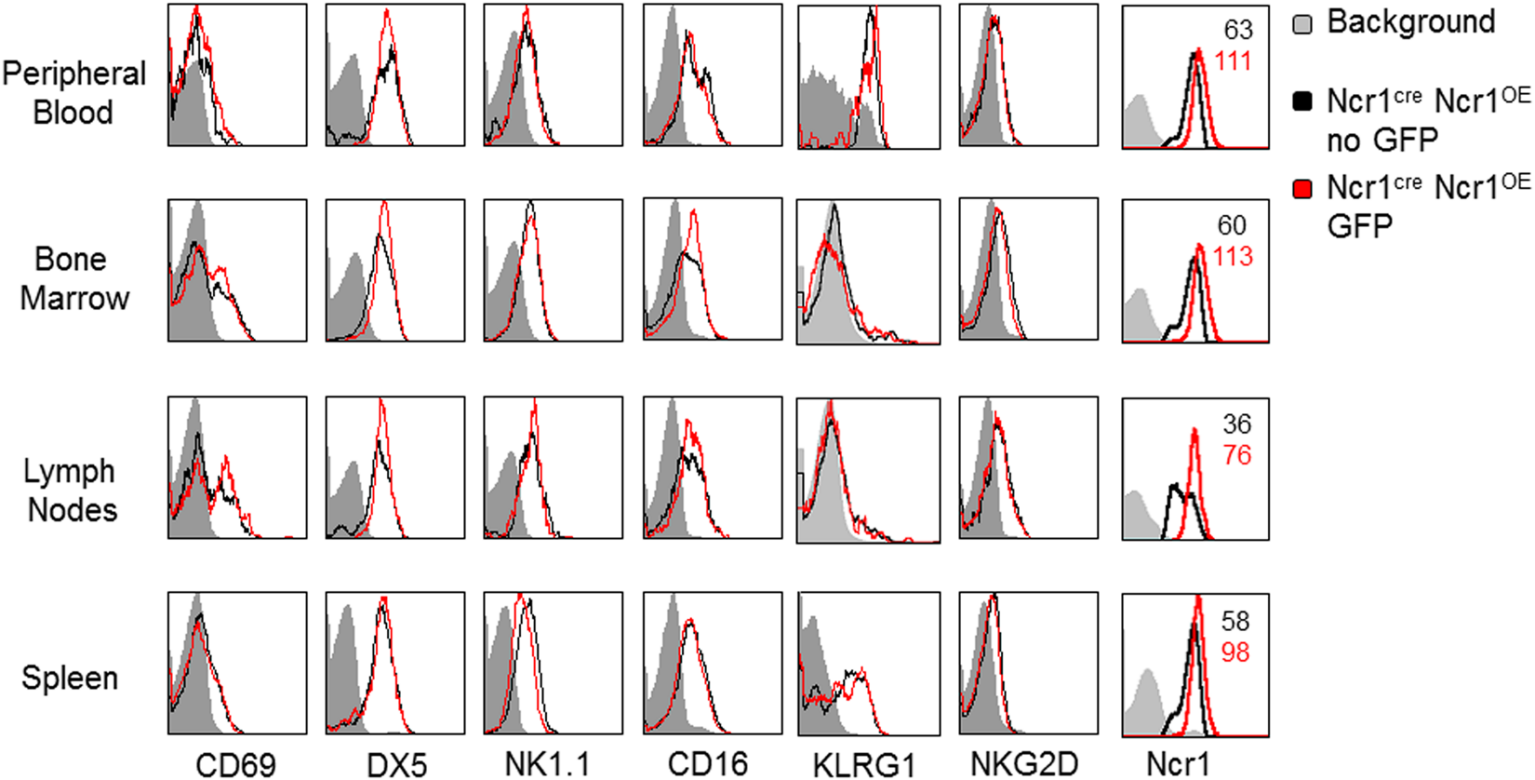
Characterization of NK cell receptors expressed by Ncr1 overexpressing NK cells. FACS staining of Ncr1 expressing cells for various NK cell receptors, as indicated. Gray histograms represent background control. Black histograms represent specific staining of the endogenous, non-GFP Ncr1 expressing cells. Red histograms represent specific staining of the overexpressed GFP positive Ncr1 expressing cells. Where differences were observed, MFIs are indicated, each MFI matching the color of its respective histogram. Each FACS plot is representative of six transgenic mice. The figure combines data from three independent experiments.

In a parallel attempt, we crossed the Rosa26^*Flox-Stop-Flox-IRES-Ncr1*^ mice with Ncr1^cre^ mice^47^, generating offspring denoted Ncr1^cre^ Ncr1^OE^. Ncr1^cre^ Ncr1^OE^ mice were born at Mendelian ratios with no obvious phenotypic abnormalities. Careful pathological analysis indicated no potential developmental abnormalities or signs of autoimmunity (Figure S1). To assess Ncr1 expression in the Ncr1^cre^ Ncr1^OE^ mice, peripheral blood lymphocytes of littermate controls, and Ncr1^cre^ Ncr1^OE^ transgenes (TG) were stained for Ncr1. Interestingly, we observed a mosaic overexpression of Ncr1 in Ncr1^cre^ Ncr1^OE^ mice, i.e, around two thirds of the NK cells overexpress Ncr1 along with GFP, while the rest expresses only the endogenous Ncr1 (Fig. 2A and B). To verify that Ncr1 over expression is restricted to NK cells, we sorted Peripheral Blood Lymphocytes (PBLs) of Ncr1^cre^ Ncr1^OE^ mice, on the basis of GFP expression. We stained these two populations with anti CD3 and anti CD19 antibodies, and observed no GFP expression in the CD3+ or CD19+ cells and no expression of CD3 or CD19 in the GFP positive cells (Fig. S2A). When staining for Ncr1 we observed over expression only in the GFP+ cells which are CD3 and CD19 negative (Fig. S2B). The increased expression of Ncr1 in the Ncr1^cre^ Ncr1^OE^ NK cells was evident when compared to the endogenous NK cell population within the same Ncr1^cre^ Ncr1^OE^ mice (Fig. 2A) or to littermates (Fig. 2A and B). We next assessed the Ncr1 expression in various tissues, including peripheral blood, bone marrow, lymph nodes and spleen. In all sites, NK cells displayed a mosaic Ncr1 expression. There was a consistent correlation between GFP positivity and Ncr1 overexpression (Fig. 2C, quantified in 2D). The enforced expression of Ncr1 was not accompanied by an altered expression of various NK cell receptors when comparing GFP^+^ and GFP^−^ NK cells derived from Ncr1^cre^ Ncr1^OE^ mice (Fig. 3).

### Ncr1^cre^ Ncr1^OE^ NK cells display enhanced killing of influenza

**Figure 4:**
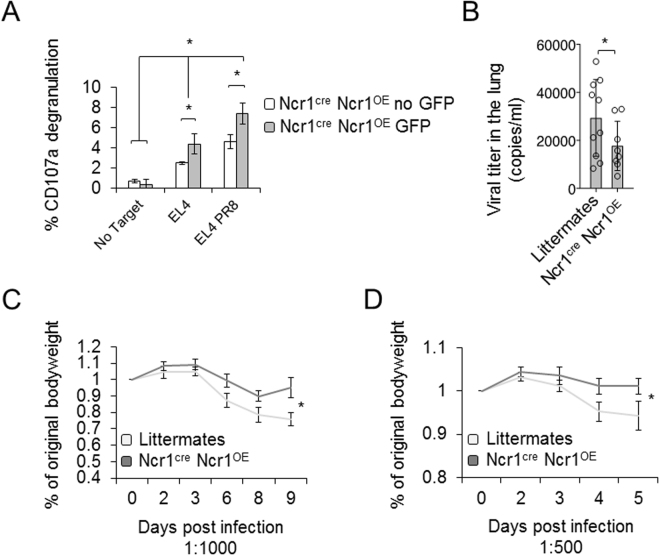
Ncr1^cre^ Ncr1^OE^ improved control of influenza *in vitro* and *in vivo*. (**A**) Isolated NK cells were sorted for GFP and incubated with the indicated targets at 1:1 ratio. CD107a degranulation was assessed. The experiment was conducted three times, including 15 mice. The figure shows one representative experiment. Values are shown as mean ± SD. *P < 0.05. (**B**) Ncr1^cre^ Ncr1^OE^ TGs were infected with influenza virus (1:500). Lungs were harvested at day 5 post infection and viral titer was quantified. 10 littermates and 8 Ncr1^cre^ Ncr1^OE^ TGs were used. Values are shown as mean ± SEM. *P < 0.05. (**C,D**) Littermates and Ncr1^cre^ Ncr1^OE^ TGs were infected with two doses of PR8 influenza and weight loss was monitored daily. The figure is representative of three independent experiments including 15 transgenic mice and at least 15 littermates.

Influenza virus resistance is the hallmark of NK cell immunity against pathogens and the HA of influenza virus was the first Ncr1 ligand identified^38^. To test whether the NK cells in the transgenic mice kill influenza virus infected cells more efficiently, we isolated NK cells from BPLs using negative selection and sorted the Ncr1 expressing cells based on GFP expression. We collected equal numbers of the endogenous GFP^−^ Ncr1 expressing cells and GFP^+^, Ncr1 overexpressing cells. These two cell populations were then, co-cultured with either untreated EL4 cells, or EL4 cells incubated with PR8 influenza virus. In line with published literature, uninfected EL4 cells alone induced activation of the NK cells, as EL4 cells express an unknown cellular ligand for Ncr1. NK cell degranulation was significantly increased upon incubation with influenza virus infected EL4 cells (Fig. 4A). Ncr1 overexpressing cells (identified by GFP and increased Ncr1 MFI), were significantly better killers as compared to cells with endogenous Ncr1 expression. This was true when killing EL4 cells alone, or in the presence of influenza virus (Fig. 4A).

Next, we infected littermates and Ncr1^cre^ Ncr1^OE^ mice with PR8 influenza virus and assessed the level of viral load in the lungs. We found that viral lung clearance was significantly better in Ncr1^cre^ Ncr1^OE^ mice compared to littermate mice (Fig. 4B). We also inoculated littermates and Ncr1^cre^ Ncr1^OE^ mice with two doses of PR8 influenza virus, and monitored body weight daily. In both doses, all animals started to lose weight shortly after inoculation, albeit with different kinetics. Littermates started to lose weight before the Ncr1^cre^ Ncr1^OE^ TGs, and their weight loss was significantly more pronounced (Fig. 4C and D).

### Ncr1^cre^ Ncr1^OE^ mice better control tumor metastases

**Figure 5:**
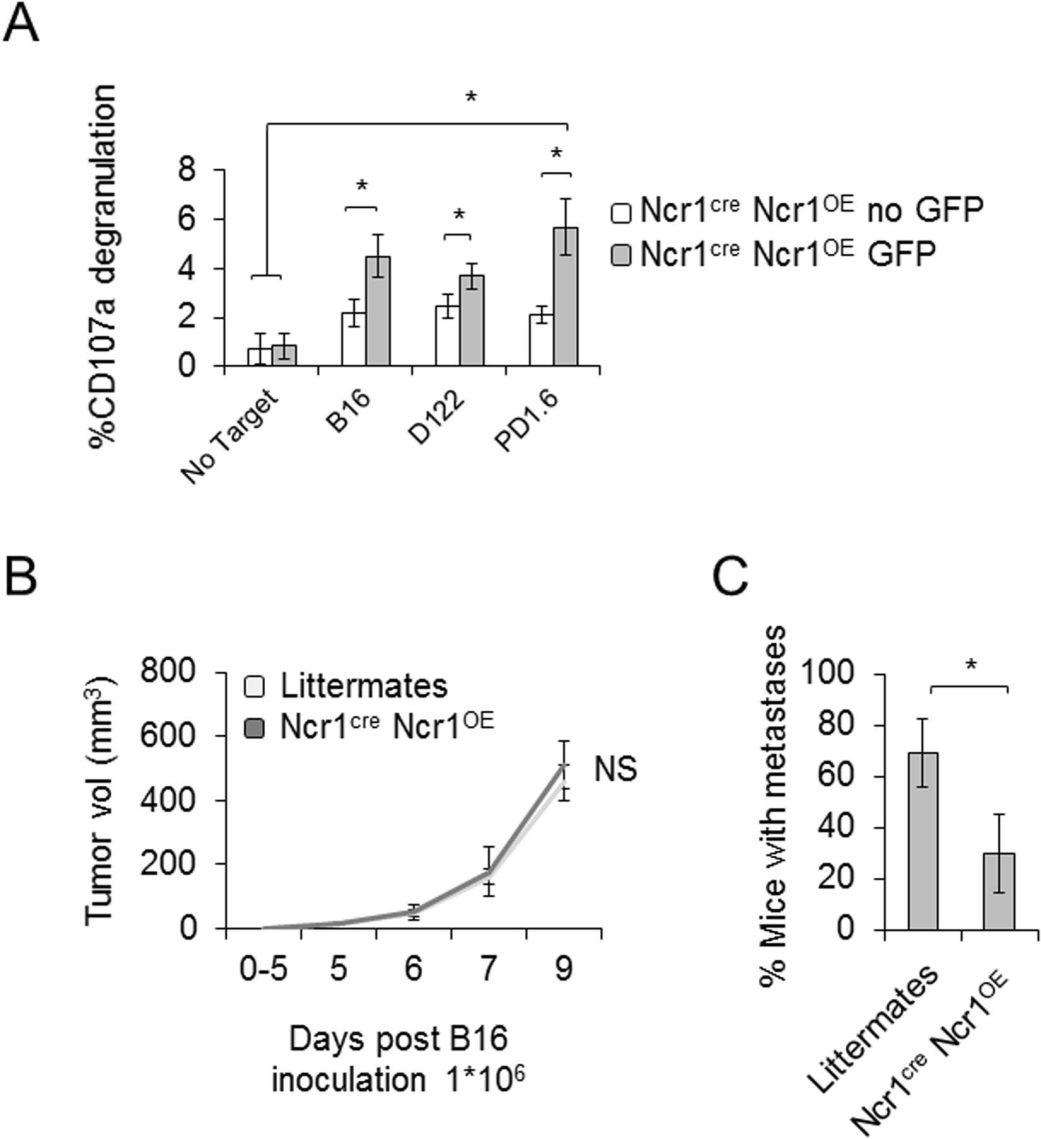
Ncr1^cre^ Ncr1^OE^ improved control of tumors and metastases *in vitro* and *in vivo*. (**A**) Isolated NK cells were sorted for GFP and incubated with the indicated targets at 1:1 ratio. CD107a degranulation was assessed using flow cytometry. The experiment was conducted three times, including 15 mice. The figure shows one representative experiment. Values are shown as mean ± SD. *P < 0.05. (**B**,**C**) Littermates and Ncr1^cre^ Ncr1^OE^ TGs were inoculated with B16 cells to the flank, and tumor growth was monitored daily (**B**). Peritoneal metastases were assessed within each animal when tumors reached endpoint volumes (1000 mm^3^) and no later than day 14 post tumor inoculation (**C**). The figure is representative of three independent experiments including 15 transgenic mice and at least 15 littermates.

We next extended our study to NK cell mediated control of tumors. We again isolated NK cells and separated GFP^−^ and GFP^+^ Ncr1 expressing cells. Different tumor lines including B16, D122 and PD1.6 were used as they all express an unidentified Ncr1 ligand^16,17,20^. Ncr1 overexpressing cells exhibited higher degranulation levels upon challenge with the different tumor cells, compared to the GFP^−^ cells (Fig. 5A).

*In vivo* studies confirmed these results; Ncr1^cre^ Ncr1^OE^ NK mice and littermate controls were subcutaneously inoculated with B16 cells. Tumor growth and final tumor volume were comparable between the Ncr1^cre^ Ncr1^OE^ mice and the littermate controls (Fig. 5B). Importantly, when tumors reached end-points, significantly lower metastases were observed in the Ncr1^cre^ Ncr1^OE^ mice compared to their littermates (Fig. 5C).

## Discussion

We live in an age where antibiotics resistance is a major threat to modern society, and where the greatest successes in cancer therapy were achieved not only by conventional chemotherapy but also by harnessing the immune system, using checkpoint receptor inhibitors^48,49^ and the adoptive transfer of genetically modified T cells^50^.

Here we generated a mouse (denoted Rosa26^*Flox-Stop-Flox-IRES-Ncr1*^) that enables the overexpression of Ncr1, one of the most important NK cell receptors, in any given cell lineage. We initially wanted to overexpress Ncr1 in T cells, with the idea in mind to generate cells with enhanced killing ability, having both innate and adoptive receptors. However, interestingly, Ncr1 was not expressed on the cell surface of the T cells in these mice. Both CD2 and LCK are not exclusive markers for T cells as they are also expressed in cells and to some extent in B cells (http://www.immgen.org/databrowser ^51,52^). While some GFP expression was observed in CD19 positive B cells, Ncr1 was not detected in these cells either, in both mice models. Why Ncr1 was not expressed on the surface of T or B cells of CD2 ^cre^ or LCK^cre^ mice is an interesting question, which might have relevance to the fact that Ncr1 is a specific and almost exclusive marker of NK cells. In contrast, in NK cells Ncr1 was overexpressed. Interestingly, a mosaic phenotype of Ncr1 overexpression was evident enabling comparing NK cells overexpressing Ncr1 and normal Ncr1 expressing NK cells within the same mouse.

We next went on to explore the prospect of utilizing NKp46/Ncr1 overexpression in therapy. We used two hallmark NKp46/Ncr1 related challenges to test the Ncr1 overexpressing NK cells function *in vitro*, and *in vivo*. The influenza virus HA was the first ligand discovered for NKp46/Ncr1^39^, and it was demonstrated repeatedly that Ncr1 deficient mice are more susceptible to influenza virus infection^6–9,18^ than Ncr1 sufficient WT mice. Indeed, we demonstrate that Ncr1 overexpression endows better protection from influenza virus infection, corroborating the role of Ncr1 in influenza virus recognition and elimination, and highlighting the possibility of manipulating NKp46 as successful treatment for influenza virus and other viral infections.

NK cells are particularly promising agents in immunotherapy, as they are capable of recognizing and eradicating various cancers efficiently, however are not subject to many complications that may obstruct T cell based immunotherapies, such as GVHD, or uncontrolled secretion of cytokines^53–56^. With that regard, as we and others previously demonstrated^17,57^, we now affirm the strong involvement of NKp46 in controlling tumor metastases, as the Ncr1 over expressing mice better control metastases formation.

Around 5 years ago, another NKp46 related mouse was generated by N-ethyl-N-nitrosourea (ENU) mutagenesis, denoted Noé^58^ where a single point mutation in Ncr1 transformed the tryptophan in position 32 to arginine (W32R). In these mice Ncr1 expression appeared to be absent from the cell surface. As the Noé mice exhibited a hyperactive phenotype against various challenges, some questions were raised regarding the involvement of Ncr1 in NK cell education. However, this hyperactive phenotype was not observed in *bona fide* Ncr1 knockout mice^6,7,9,14,16,18,19,40,41,46^, questioning the involvement of NKp46/Ncr1 in NK cell education. We later demonstrated that NKp46/Ncr1 W32R could be expressed on the cell surface, that it is aberrantly glycosylated, and that the aberrant expression of NKp46/Ncr1, rather than its absence, is probably responsible for the NKp46/Ncr1 W32R hyperactivated phenotype^19,59^. The results presented here demonstrating enhanced Ncr1-dependent activities following overexpression of Ncr1 further support this conclusion.

In light of the results presented here, we propose that the overexpression of NKp46 in NK cells may offer a promising new avenue for the treatment of cancer metastases and viral diseases.

## Methods

### Mice

#### Generation of the Rosa26^*Flox-Stop-Flox-IRES-Ncr1*^

Cloning the Ncr1 into the STOP-eGFP-ROSA26TV vector: Ncr1 was amplified by PCR using C57BL/6 mRNA, isolated using R1055 Quick-RNA MiniPrep Kit (Eisenberg bros, Israel) and reverse transcribed to cDNA using M-MLV Reverse Transcriptase 28025–013 (Invitrogen, ThermoFisher Scientific, MA). Primers for cloning the Ncr1 to STOP-eGFP-ROSA26TV were: Ncr1 5′ including AcsI tt GGCGCGCC gcc gcc acc atg ctg cca aca ctc act gc; Ncr1 3′ including AcsI ttGGCGCGCC tcacaaggccccaggagttg.

Amplified Ncr1 cDNA was cloned into the AcsI site of STOP-eGFP-ROSA26TV (Addgene 11739). To verify expression of the construct, it was expressed in HEK293T cells and staining for Ncr1 verified cell surface expression.

SB for detection of positive ES clones: The resulting vector was linearized and electroporated into V6.5 mouse ES cells. After selection with G418 individual clones were isolated and expanded. Genomic DNA from these clones was cut with Mfe1 and Southern Blot was done using internal probe covering the first 300 bp of NCR cDNA as well as 5′ external probe for mouse Rosa locus. DNA was transferred to a nitrocellulose membrane that was next hybridized with a radioactively labeled probe and developed using enhanced chemiluminescent substrate (Thermo Scientific). 5′ probe 8613–9128.

Homology arm 10137–5′. Insert (neo + stop + Ncr1 IRES GFP) − 11161–16643. Homology arm 20983–3′. 3′ probe – 21290–21820. Internal probe – 13823–14113.

Ncr1 Expression in ES cells following cre treatment: Southern Blot confirmed single and specific insertion in about 60% of the analyzed clones. These positive clones were treated with TAT-cre protein (Excellgene), and marked elevation of Ncr1 expression was observed using qPCR.

Generation of Ncr1 Rosa Stop chimeras: ES cells were injected to blastocyst stage embryos, which were subsequently transferred to foster mothers. The resulting chimeras were crossed to WT C57BL/6 mice to yield progeny carrying the desired genomic modification. The mice were genotyped using the following primers: R26_stop_F2CCACACAGGCATAGAGTGTCTGCTATTAA. R26_stop_R2TTGTGACCAT GATGCTGGGTTTG. Product 535. Ncr1 was amplified from 3 Rosa26Flox-Stop-Flox-IRES-Ncr1 offspring genomic DNA and sent for sequencing, which verified no mutations occurred. DNA was isolated using DNeasy Blood & Tissue Kit (Qiagen 69504).

Generation of Ncr1^cre^ Ncr1^OE^ mice: Ncr1^cre^ Ncr1^OE^ mice were generated by crossing Rosa26^*Flox-Stop-Flox-IRES-Ncr1*^

With Ncr1^cre^ mice^47^. All the experiments were performed in a specific pathogen free unit of the Hebrew University Medical School (Ein-Kerem, Jerusalem). All experimental protocols and procedures were approved by and performed in accordance with the guidelines of the Hebrew University Medical School ethics committee (MD-12–13471–5).

mAbs, CD107 assay flow cytometry and Cells: mAbs used where anti mouse NKp46 (BioLegend 137608 clone 29A1.4), CD3 (BioLegend 100312 clone 145–2C11), CD4 (BioLegend 100412 clone GK1.5) CD8 (BioLegend 100712 clone 53–6.7), CD69 (BioLegend 104508 clone H1.2F3) CD107a (BioLegend 121614 clone 1D4B), DX5 (BioLegend 108908 clone DX5), NK1.1 (BioLegend 108708 clone PK136), CD16 (BioLegend 101326 clone 93), KLRG1 (BioLegend 138412 clone 2F1/KLRG1) and NKG2D (BioLegend 130212 clone CX5). For assessing degranulation PB NK cells were enriched using EasySep™ negative selection (STEMCELL) and sorted (Aria, BD). CD107a degranulation was performed using equal numbers of Ncr1 + GFP- and Ncr1 + GFP + NK cells, incubated (separately) with targets at a ratio of 1:1. Cells used in these study were B16, EL4, D122, PD1.6.

### Pathology

Samples of the major anatomical systems were embedded in paraffin, cut, stained with Hematoxylin and Eosin (H&E) and examined by a pathologist.

### Influenza and tumor development

Propagation of the human influenza virus A/Puerto Rico/8/34 H1N1 (PR8) and *in vivo* inoculation were performed as previously described^9^. In brief, mice were intranasally inoculated with PR8 influenza (1U:500 or 1U:1000) in 40ul PBS and weighed daily thereafter. Viral loads in the lungs were determined by qRT-PCR. For B16 tumor inoculation, B16 cells (1 × 10^6^) were subcutaneously injected and tumor incidence and volume were monitored as soon as visible tumors appeared using a caliber. When tumors reached a volume of around 1000 mm^3^ mice were sacrificed and their peritoneal and chest cavities were visually inspected. The presence or absence of metastases was assessed by eye. If metastatic foci were observed the tumor was considered metastatic and the mouse was considered positive for metastases. Percent of mice with metastases where evaluated.

### Statistical Analysis

Analysis of variance (ANOVA) or Student’s’ T test were performed to evaluate group differences. For all comparisons, P < 0.05 was considered significant.

### Data availability

All data generated or analyzed during this study are included in this published article (and its Supplementary Information files).

## Electronic supplementary material

Supplementary Figures
