# Editorial Expression of Concern: Increased NK cell immunity in a transgenic mouse model of NKp46 overexpression

**DOI:** 10.1038/s41598-026-39507-2

**Published:** 2026-02-17

**Authors:** Ariella Glasner, Batya Isaacson, Sergey Viukov, Tzahi Neuman, Nehemya Friedman, Michal Mandelboim, Veronika Sexl, Jacob H. Hanna, Ofer Mandelboim

**Affiliations:** 1 The Lautenberg Center for General and Tumor Immunology, Department of Immunology and Cancer Research, IMRIC, Faculty of Medicine, The Hebrew University Medical School, Jerusalem, Israel; 2 Department of Molecular Genetics, Weizmann Institute of Science, 7610001 Rehovot, Israel; 3 Department of Pathology, Hadassah Medical Organization, The Hebrew University Medical Center, Jerusalem, Israel; 4 Central Virology Laboratory, Ministry of Health, Chaim Sheba Medical Center, Tel-Hashomer, Ramat-Gan, Israel; 5 Department of Epidemiology and Preventive Medicine, School of Public Health, Sackler Faculty of Medicine, Tel-Aviv University, Tel-Aviv, Israel; 6 Institute of Pharmacology and Toxicology, University of Veterinary Medicine, Vienna, Austria

Editorial Expression of Concern to: *Scientific Reports* 10.1038/s41598-017-12998-w, published online 12 October 2017

The Editors are issuing an Editorial Expression of Concern to alert readers that concerns were raised regarding similarities within the FACS plots in Figure 1E and Supplementary Figure 2A of this Article. The Authors provided explanations for the matters raised; however, the original data were no longer available. As such, it was not possible to determine whether the descriptions of these figures are accurate. Readers are therefore advised to interpret the data presented in Figure 1E and Supplementary Figure 2A with caution.

Ariella Glasner, Batya Isaacson, Michal Mandelboim and Ofer Mandelboim disagreed with this Expression of Concern; Sergey Viukov and Jacob H. Hanna did not explicitly state their agreement or disagreement; Tzahi Neuman, Nehemya Friedman and Veronika Sexl did not respond to correspondence from the Editor.

