# Supplementary material for: Increased NK cell immunity in a transgenic mouse model of NKp46 overexpression
Source: Sci Rep. 2017 Oct 12;7:13090. doi: 10.1038/s41598-017-12998-w (PMC5638832; doi:10.1038/s41598-017-12998-w)
Supplement: Supplementary file 1 — Supplementary Figures [file 41598_2017_12998_MOESM1_ESM.pdf]

Increased NK cell immunity in a transgenic mouse model of NKp46 overexpression

Ariella Glasner[1], Batya Isaacson[1], Sergey Viukov[2], Tzahi Neuman[3], Nehemya Friedman[4,5]
Michal Mandelboim[4,5], Veronika Sexl[6], Jacob H Hanna[2] and Ofer Mandelboim[1]

[1]The Lautenberg Center for General and Tumor Immunology, Department of Immunology and Cancer Research, IMRIC, Faculty of Medicine, The Hebrew University Medical School, Jerusalem, Israel.

[2]Department of Molecular Genetics, Weizmann Institute of Science, Rehovot 7610001, Israel.

[3]Department of Pathology, Hadassah Medical Organization, The Hebrew University Medical Center, Jerusalem, Israel.

[4]Central Virology Laboratory, Ministry of Health, Chaim Sheba Medical Center, Tel-Hashomer, Ramat-Gan, Israel

[5]Department of Epidemiology and Preventive Medicine, School of Public Health, Sackler Faculty of Medicine, Tel-Aviv University, Tel-Aviv, Israel

[6]Institute of Pharmacology and Toxicology, University of Veterinary Medicine, Vienna, Austria.

Correspondence should be addressed to Ofer Mandelboim**:** oferm@ekmd.huji.ac.il

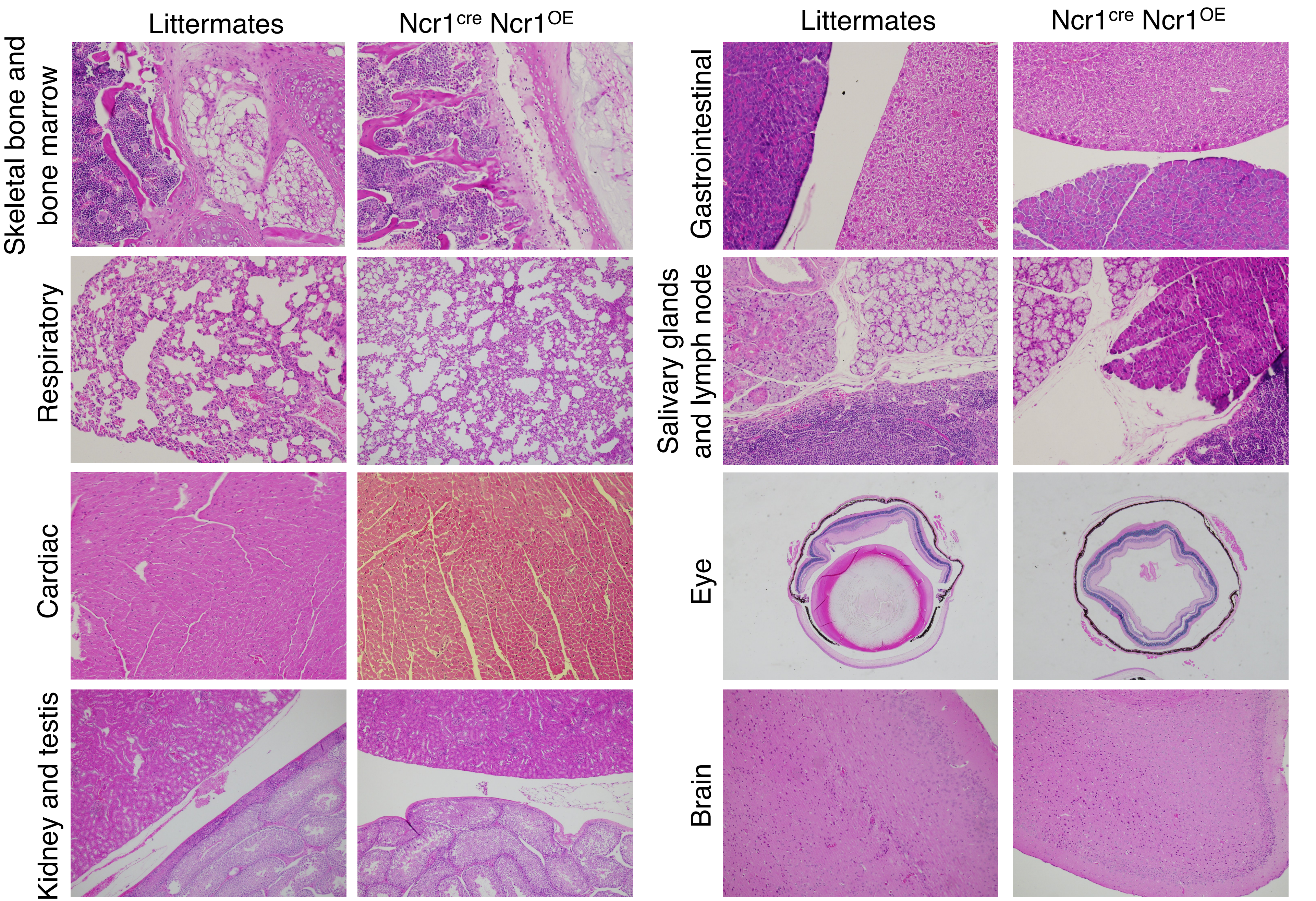

Figure S1. Comparison of the major anatomical systems between Ncr1cre Ncr1OE and littermate controls

Samples of the major anatomical systems representative of three Ncr1^{cre} Ncr1^{OE} TGs and three littermates are shown. 100 or 200 fold magnification was used.

Figure S1

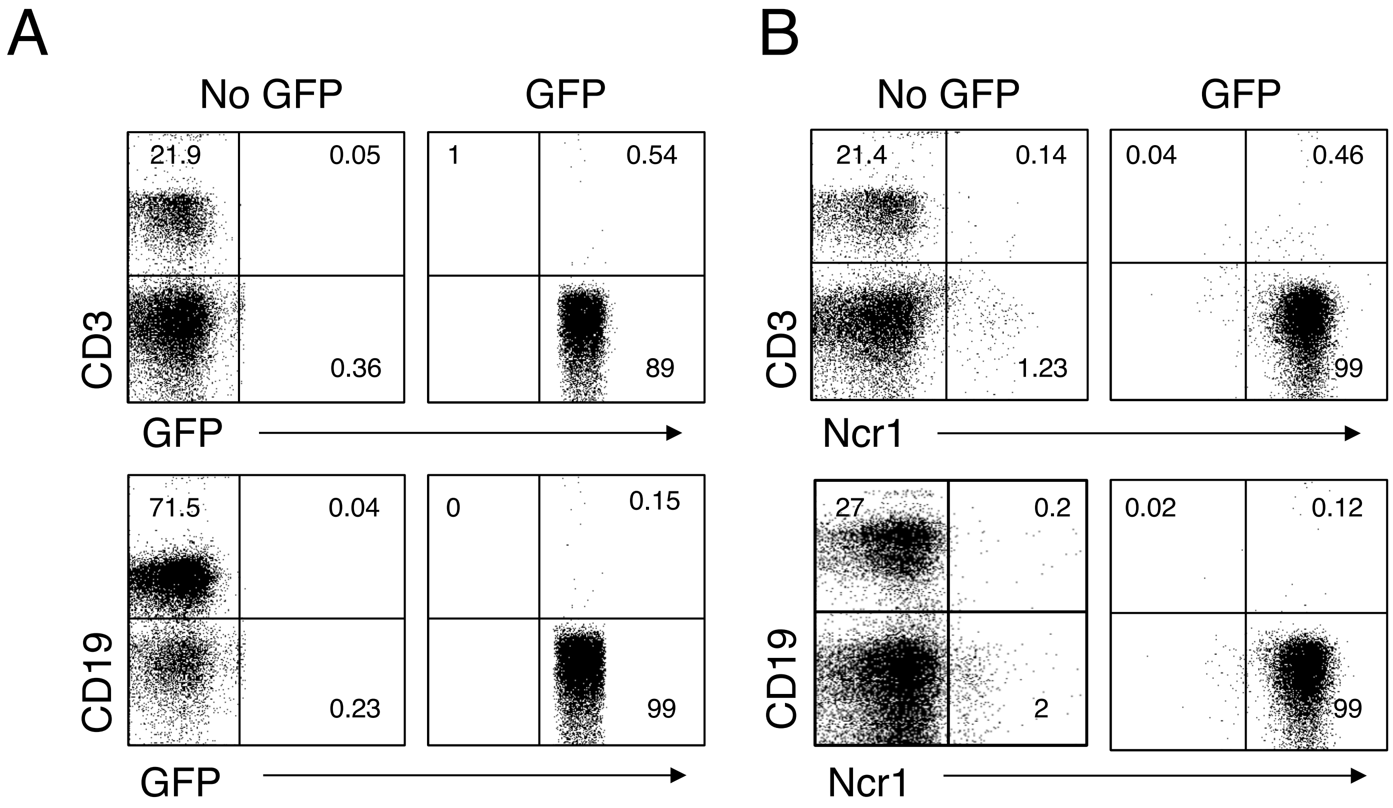

Figure S2. GFP or Ncr1 are not expressed on non-NK lymphocytes

(A,B) FACS plots depicting CD3 and CD19 expression in GFP (A) or Ncr1 (B) expressing cells in GFP expressing lymphocytes, compared to non-GFP expressing lymphocytes. The plots are representative of two independent stainngs. Percentages are indicated.

Figure S2
